# Safety and Efficacy of Cannabis in Autism Spectrum Disorder

**DOI:** 10.15844/pedneurbriefs-34-25

**Published:** 2020-12-24

**Authors:** Abhinaya Ganesh, Safiullah Shareef

**Affiliations:** 1 The University of North Texas Health Science Center- Texas College of Osteopathic Medicine, Fort Worth, TX; 2 Texas Child Neurology, Plano, TX

**Keywords:** Autism, Cannabis, Cannabidiol, Neurodevelopment, CBD, THC, ASD, Neurology, Pediatrics

## Abstract

Investigators from the Soroka University Medical Centre, The Hebrew University of Jerusalem, and Tikun Olam Ltd. in Israel studied the safety and efficacy of medical cannabis treatment on 188 patients with autism spectrum disorder (ASD) for six months.

Investigators from the Soroka University Medical Centre, The Hebrew University of Jerusalem, and Tikun Olam Ltd. in Israel studied the safety and efficacy of medical cannabis treatment on 188 patients with autism spectrum disorder (ASD) for six months. The study examined the efficacy of cannabidiol (CBD) through a quality-of-life assessment, global assessment rating, and an ASD symptom severity assessment looking at restlessness, rage attacks, agitation, speech impairment, cognitive impairment, anxiety, incontinence, depression, and more. The safety of CBD was examined by assessing physiological and cognitive side effects. The largest national provider of medical cannabis in Israel employed several of the authors and sponsored this study. [1]

COMMENTARY. ASD is a developmental disorder with two clusters of symptoms: 1. persistent deficits in social communication and social interaction, and 2. restricted, repetitive patterns of behavior, interests, or activities [2]. Pharmacological treatment in ASD focuses on attenuating comorbid symptoms. Recent studies and anecdotal evidence have contributed to the increasing popularity of cannabidiol as a possible addition to current treatment regimens. Although the exact mechanism is unknown, cannabidiol is hypothesized to target a dysfunctional endocannabinoid system [3] and effect oxytocin release during social interaction [4]. Prior studies have looked at CBD's utility in treating hyperactivity, sleep, self-injury, anxiety, and other behavioral issues [5]. This study arrives at similar conclusions supporting the potential therapeutic utility of CBD.

Safety was assessed by analyzing the frequency of side effects, while efficacy was analyzed using the ASD symptom severity assessment. Many of the same symptoms were reported as both side effects of the treatment and comorbid symptoms of prior diagnoses, making it difficult to interpret the results. The safety of medical cannabis was assessed within a one-month, and six-month follow up. Progress of patients who received extra THC doses was not differentiated from the remainder of patients.

Long-term safety from prolonged usage could not be determined from this study. Patient outcomes and side effects were only recorded at months one and six; the selection of this specific follow-up period is not clearly explained. Observational studies, such as this one, are essential in identifying the short-term safety and efficacy of medications; CBD's long-term safety for ASD spectrum patients is unclear.

Subjective self-reporting by parents of patients was used as the basis of this study. Parents' expectations likely influenced their reporting. Assessment of parental perceptions and expectations of safety and efficacy at baseline could have elucidated these potentially confounding factors. Furthermore, this study was sponsored by a large local CBD manufacturer and the study population was using the product, which could have added an extra layer of parental reporting bias. Prospective, independent, placebo-controlled clinical trials are required to determine more accurate long-term efficacy and dosing guidelines for CBD in ASD.

## Disclosures

The authors have declared that no competing interests exist.

## References

[1] Bar-Lev Schleider L, Mechoulam R, Saban N, Meiri G, Novack V (2019). Real life Experience of Medical Cannabis Treatment in Autism: Analysis of Safety and Efficacy. Sci Rep.

[2] American Psychiatric Association (2013). Diagnostic and statistical manual of mental disorders.

[3] Agarwal R, Burke SL, Maddux M (2019). Current state of evidence of cannabis utilization for treatment of autism spectrum disorders. BMC Psychiatry.

[4] Wei D, Dinh D, Lee D, Li D, Anguren A, Moreno-Sanz G (2016). Enhancement of Anandamide-Mediated Endocannabinoid Signaling Corrects Autism-Related Social Impairment. Cannabis Cannabinoid Res.

[5] Barchel D, Stolar O, De-Haan T, Ziv-Baran T, Saban N, Fuchs DO (2019). Oral Cannabidiol Use in Children With Autism Spectrum Disorder to Treat Related Symptoms and Co-morbidities. Front Pharmacol.

